# Contrasting Disease Progression, Microglia Reactivity, Tolerance, and Resistance to *Toxoplasma gondii* Infection in Two Mouse Strains

**DOI:** 10.3390/biomedicines12071420

**Published:** 2024-06-26

**Authors:** Daniel G. Diniz, Jhonnathan H. P. de Oliveira, Luma C. F. Guerreiro, Gabriel C. de Menezes, Alexa C. L. de Assis, Tainá Q. Duarte, Izabelly B. D. dos Santos, Flávia D. Maciel, Gabrielly L. da S. Soares, Sanderson C. Araújo, Felipe T. de C. Franco, Ediclei L. do Carmo, Rafaela dos A. B. Morais, Camila M. de Lima, Dora Brites, Daniel C. Anthony, José A. P. Diniz, Cristovam W. P. Diniz

**Affiliations:** 1 Laboratório de Investigações em Neurodegeneração e Infecção, Instituto de Ciências Biológicas, Hospital Universitário João de Barros Barreto, Universidade Federal do Pará, Belém 66073-005, Pará, Brazil; danielguerreirodiniz@gmail.com (D.G.D.); jhonnathan.oliveira@ics.ufpa.br (J.H.P.d.O.); lumacristinabiologa@gmail.com (L.C.F.G.); gabrielmenezes.9828@gmail.com (G.C.d.M.); alexacamila15@gmail.com (A.C.L.d.A.); quintellataina@gmail.com (T.Q.D.); ibiase24@gmail.com (I.B.D.d.S.); flavia100maciel@gmail.com (F.D.M.); gabriellylis@gmail.com (G.L.d.S.S.); camilamendesdelima@gmail.com (C.M.d.L.); 2 Laboratório de Microscopia Eletrônica, Instituto Evandro Chagas, Belém 66077-830, Pará, Brazil; sandersonaraujo@iec.gov.br (S.C.A.); felipefranco@iec.gov.br (F.T.d.C.F.); joseantonio@iec.gov.br (J.A.P.D.); 3 Núcleo de Pesquisas em Oncologia, Hospital Universitário João de Barros Barreto, Universidade Federal do Pará, Belém 66075-110, Pará, Brazil; 4 Laboratório de Biologia Molecular e Neuroecologia, Instituto Federal do Pará, Campus Bragança, Bragança 68600-000, Pará, Brazil; 5 Seção de Parasitologia, Instituto Evandro Chagas, Belém 67030-000, Pará, Brazil; edicleicarmo@iec.gov.br (E.L.d.C.); rafaelamorais@iec.gov.br (R.d.A.B.M.); 6 Research Institute for Medicines (iMed.ULisboa), Faculty of Pharmacy, Universidade de Lisboa, 1649-003 Lisbon, Portugal; dbrites@ff.ulisboa.pt; 7 Department of Pharmaceutical Sciences and Medicines, Faculty of Pharmacy, Universidade de Lisboa, 1649-003 Lisbon, Portugal; 8 Laboratory of Experimental Neuropathology, Department of Pharmacology, University of Oxford, Oxford OX1 2JD, UK; daniel.anthony@pharm.ox.ac.uk

**Keywords:** dentate gyrus, microglia, ocular infection, resistance, sickness behavior, tolerance, *Toxoplasma gondii*

## Abstract

Our study investigated the innate immune response to *Toxoplasma gondii* infection by assessing microglial phenotypic changes and sickness behavior as inflammatory response markers post-ocular tachyzoite instillation. Disease progression in Swiss albino mice was compared with the previously documented outcomes in BALB/c mice using an identical ocular route and parasite burden (2 × 10^5^ tachyzoites), with saline as the control. Contrary to expectations, the Swiss albino mice displayed rapid, lethal disease progression, marked by pronounced sickness behaviors and mortality within 11–12 days post-infection, while the survivors exhibited no apparent signs of infection. Comparative analysis revealed the *T. gondii*-infected BALB/c mice exhibited reduced avoidance of feline odors, while the infected Swiss albino mice showed enhanced avoidance responses. There was an important increase in microglial cells in the dentate gyrus molecular layer of the infected Swiss albino mice compared to the BALB/c mice and their respective controls. Hierarchical cluster and discriminant analyses identified three microglial morphological clusters, differentially affected by *T. gondii* infection across strains. The BALB/c mice exhibited increased microglial branching and complexity, while the Swiss albino mice showed reduced shrunken microglial arbors, diminishing their morphological complexity. These findings highlight strain-specific differences in disease progression and inflammatory regulation, indicating lineage-specific mechanisms in inflammatory responses, tolerance, and resistance. Understanding these elements is critical in devising control measures for toxoplasmosis.

## 1. Introduction

*Toxoplasma gondii* is a ubiquitous parasitic protozoan known to infect a wide range of warm-blooded animals, including humans. The prevalence of chronic *T. gondii* infections is notably high worldwide, with significant regional disparities. African countries exhibit the highest prevalence value at 61.4%, followed by Oceania (38.5%), South America (31.2%), Europe (29.6%), the U.S.A./Canada (17.5%), and Asia (16.4%) [1]. These numbers underscore the substantial burden of *T. gondii* infections globally, impacting budget allocations for the treatment of both acute and chronic cases [2,3]. Moreover, individuals of all ages are susceptible to these infections [4], with heightened risks for immunocompromised individuals, while for transplant recipients, it can be fatal [5].

### 1.1. Host’s Immune Response, Tolerance, and Resistance

Upon infection, the immune system of the host is mobilized to restore homeostasis and combat the invading pathogen. The immune response can either present as resistance, aiming to eliminate the infectious agent, or as disease tolerance, aiming to alleviate the harmful impacts of the infection [6,7]. The innate immune system in particular plays a pivotal role in both resistance and disease tolerance by recognizing pathogen-associated molecular patterns (PAMPs) and then coordinating the inflammatory response [8]. Experimental studies employing murine models have shed light on *T. gondii*’s evasion strategies against the host immune system and have emphasized the interplay between parasite virulence factors and host immune pathways [9,10,11]. While acute infections are typically controlled, chronic infections may persist, leading to cyst formation, particularly within the brain [12,13]. Thus, disease progression and the associated neuropathology are inextricably linked to the host immune response and highlight the central role of inflammatory modulation [14,15,16], with *T. gondii* effectors contributing to the regulation of host inflammatory responses [17].

### 1.2. Sickness Behavior and Microglial Response

Sickness behavior, encompassing a range of behavioral changes in response to infections, is an adaptive strategy mediated by pro-inflammatory cytokines [18,19]. In murine models, the acute infection-induced sickness behavior is characterized by observable clinical signs, such as altered posture, anhedonia, and increased immobility time, underpinned by the systemic molecular and cellular response to infection [20]. At the same time, long-term behavioral alterations are associated with sustained neuroinflammation (e.g., Iba1 microglial immunoreactivity and inflammatory cerebral perivascular cuffs) [21,22]. Microglia make up around 7% of the non-neuronal cells in various brain structures and in the entire brain of all mammalian species that have been studied so far [23]. Despite their small percentage, microglia are known for their diverse phenotype and important contributions to maintaining the homeostasis of the central nervous system [24].

When the microglia cells respond to an injury, whether it is acute or chronic, they change their functional and morphological characteristics [25,26,27,28]. These changes seem to vary depending on the region [29,30] and the species [31,32], and they are regulated by a variety of stimuli that can occur in both healthy and diseased states [33,34,35]. Microglia cells play a crucial role in the pathological progression of all brain diseases [27], and they exhibit multiple morpho-functional characteristics in response to them. It has been demonstrated that reactive microglia increase IL-1β expression levels, and this is correlated with different morphotypes of microglia that have been identified through hierarchical cluster analysis of morphometric parameters [36,37].

Microglia, as the principal immune effectors within the brain, play a crucial role in mediating sickness behaviors and serve as indicators of neuroinflammatory responses [35,36,38], which may exert adaptive or maladaptive effects, depending on the context [39]. Investigating the influence of parasites on innate mouse behavior and olfactory preferences in this context provides valuable insights into the interplay between host behavior and immune responses [40].

### 1.3. Microglia and Toxoplasmosis

During *T. gondii* infection, immune sensors recognize parasite components, triggering the production of pro-inflammatory mediators [41]. Interferon-γ emerges as a key mediator of resistance against *T. gondii* and has a crucial role in the transition from acute to chronic infection stages [42,43]. Notably, behavioral changes associated with *T. gondii* infection are linked to distinct stages of the parasite lifecycle [44,45], with encysted bradyzoites implicated in modulating host behavior [46,47]. Microglia respond to *T. gondii* infection by producing immune mediators and interacting with lymphocytes, contributing to acquired immunity [48]. Moreover, chronic *T. gondii* infection elicits morphological and numerical changes in the microglia, associated with altered behavioral responses [49].

The complex interplay between host immune responses, sickness behavior, and microglial dynamics during *T. gondii* infection underscores the multifaceted nature of host–parasite interactions. Through investigation using experimental models, we sought to gain deeper insights into the pathogenesis of *T. gondii* and to identify potential therapeutic targets to mitigate the impact of toxoplasmosis on host health and behavior. It is essential to clarify that the conclusions drawn in this study are based on findings from experimental models. By correlating microglial reactivity with sickness behavior in two distinct mouse strains, our research seeks to elucidate the role of inflammatory regulation in host tolerance and resistance to *T. gondii* infection.

## 2. Materials and Methods

In this study, we conducted a comparative assessment of microglial response and behavioral outcomes following ocular infection induced by the *T. gondii* RH strain. We revisited raw behavioral data obtained from the BALB/c lineage [49], comparing them with behavioral data collected in the current assay from the Swiss albino lineage. Additionally, we investigated the microglial response to *T. gondii* ocular infection in the dentate gyrus of both lineages during the acute phase of infection, employing three-dimensional microscopic reconstruction to analyze cell morphology. For the BALB/c lineage, reconstructions were derived from a slide collection generated in a previous study, encompassing both the acute and chronic phases. However, as the animals from the Swiss albino lineage did not progress to the chronic phase, our comparative morphological analyses were limited to the acute phase of the disease. Female albino Swiss mice were housed in compliance with the National Institutes of Health guidelines for laboratory animal care. Ethical approval for the experimental protocol was obtained from both the Ethics Committee on Experimental Animal Research at the Institute of Biological Sciences, Federal University of Pará, Brazil (CEUA nº 7961160818), and the Ethics Committee of the Evandro Chagas Institute (Protocol No. 09/2021). All the parasite handling procedures adhered strictly to the standards and criteria mandated by the International Biosafety Committee.

### 2.1. Animal Handling, Anesthesia, and the Instillation of T. gondii into the Eye

Thirty-four-month-old albino Swiss adult female mice from the animal services unit of Instituto Evandro Chagas were used in this study. They were housed in standard laboratory cages, provided ad libitum access to water and food, and maintained in a temperature-controlled environment (23 ± 2 °C) with a 12-h light–dark cycle. Tachyzoites of the *T. gondii* RH strain was acquired from the Toxoplasmosis Laboratory of Instituto Evandro Chagas.

For the experimental procedure, the animals were anesthetized using avertin (0.08 mg/5 g of body weight). They had the conjunctival sac of their left eye instilled with either 5 µL of an infected suspension containing 48.5 × 10^6^ parasites/mL (infected group) or an equal volume of saline solution (control group). Five subjects from both the infected and control groups were sacrificed at 12 days post-infection (dpi).

### 2.2. Behavioral Testing: Open Field and Olfactory Discrimination

For each mouse, we used the contrast index to assess the relative occupation of the three compartments in the olfactory test and the periphery vs. central areas in the open field task [49,50]. This index provides a valuable method for normalizing the data. It scales the data to a standard range, typically between 0 and 1, making them easier to compare and analyze. This normalization technique also ensures that the data lie within a specific range, helping to reduce the impact of outliers and making the data more interpretable. We also measured immobility during the open field task.

- *Open Field Test*

The open field test is a method used to observe the behavior of animals in a new environment. Typically, the animals tend to stay close to the walls of the arena, which is held to be anxiety behavior, although this is a controversial topic [51]. This behavior is thought to be a defense mechanism that helps to reduce potential threats, such as predation [52].

The open field test comprised a gray polyvinyl chloride box (30 cm × 30 cm × 40 cm) that was divided digitally into equal central and peripheral regions. Each animal was placed individually in the center of the arena, and its exploratory activity was video recorded for three minutes. The ANY-maze version 7.2 (Stoelting Inc., Kiel, WI, USA) software was used to determine the distance traveled, immobility, and time spent in each region. The contrast between the time spent in the periphery and the center (C = (Tp − Tc)/(Tp + Tc), where C = contrast, Tp = time spent in the periphery, Tc = time spent in the center) was calculated. The apparatus was sanitized with 70% alcohol after each test to eliminate residual odors. Weekly open field tests were conducted in the infected and control groups to monitor behavioral changes during disease progression.

- *Olfactory Discrimination Test*

Following the previous protocol, the olfactory discrimination (O.D.) test was conducted within a three-compartmental box [49]. The experiment involved placing the mice in a test apparatus consisting of three interconnected compartments. The mice were allowed to navigate through the compartments via polyvinyl tubes. Before the experiment, the mice were acclimated to clean compartments for five minutes, and the apparatus was cleaned with 70% alcohol.

During the olfactory test, the central compartment contained clean bedding, while the lateral compartments contained spoiled bedding from the mice under examination and a mix of clean straw with feline feces. Each mouse was individually placed in the central compartment facing the wall, and its behavior was recorded for three minutes. We kept track of the amount of time spent in each compartment during the olfactory test and compared it to the total test duration. The contrast index was calculated using the formula C = (Tt − Tf)/(Tt + Tf), where Tt represents the total time and Tf represents the time spent in the compartment containing feline feces. As the contrast index decreased, indicating a greater preference for the compartment with feline feces, the amount of time spent in that specific compartment increased.

### 2.3. Histological and Immunohistochemical Procedures

Upon reaching the designated survival time, final behavioral tests were conducted before the mice underwent weighing, anesthesia via intraperitoneal administration of 2,2,2-tribromoethanol (0.15 mL/g of body weight), and subsequent transcardial perfusion. The perfusion sequence involved heparinized saline followed by 4% paraformaldehyde in 0.1 M phosphate buffer (pH 7.2–7.4). As the disease proved fatal for five Swiss albino mice at the 11th dpi, all the mice of this lineage were euthanized at 12 dpi. In contrast, all the BALB/c mice recovered from the acute stage of the disease and were euthanized at two different time points: 22 or 43 dpi. The five dead albino Swiss mice were not included in the analysis.

Serial anatomical sections (80 µm thick) were obtained using a Vibratome (MICROM, model HM 650 MK, Microm International GmbH, Walldorf, Germany) and subjected to immunolabeling using polyclonal antibodies. The brain sections underwent initial antigen retrieval in 0.2 M boric acid (pH 8.0) at 70 °C for 60 min and subsequent washing in 0.1 M saline tris buffer solution (pH 7.2–7.4). The sections were then treated with 10% casein for 60 min and incubated with the primary antibody (Anti-IBA-1 polyclonal antibody, Rabbit/Wako, code 01127991, Wako Pure Chemical Industries Ltd., Osaka, Japan) diluted in tris buffer saline (pH 7.0) at a 1:500 concentration for 72 h.

Selective immunolabeling for *T. gondii* antigens utilized polyclonal antibodies from the Laboratory of Toxoplasmosis at Instituto Evandro Chagas. These antibodies were generated via oral inoculation of the BALB/c mice with 25 µL of a suspension containing ten tissue cysts of *T. gondii* cystogenic strains (VEG) diluted in 0.9% saline. At 40–45 dpi, blood was collected, and the separated serum samples underwent modified agglutination tests for anti-*T. gondii* total antibody detection.

The same *T. gondii* antigen detection procedures were adapted to the retinas and brain sections. The brain sections underwent antigen recovery in 0.2 M boric acid solution with a pH of 9.0 at 70 °C for an hour, followed by permeabilization with 5% Triton X-100 in 0.1 M saline phosphate buffer for 5 min. A 10% casein solution in 0.1 M saline phosphate buffer was employed to minimize nonspecific labeling.

The retinas underwent collagenase treatment (0.01% in 0.1 M PBS) for 10 min at room temperature to facilitate antibody penetration. Subsequently, they were washed and immersed in a solution of 10% methanol + 3% hydrogen peroxide in 0.1 M PBS for 15 min, followed by immersion in 0.1 M PBS Triton 5% twice for 5 min each.

The brain sections and retinas underwent the Mouse-on-Mouse (M.O.M.) protocol (M.O.M. kit, Vector Laboratories, Burlingame, CA, USA) with M.O.M. IgG blocking for 1 h, primary antibody incubation for 72 h, and subsequent incubation with secondary antibodies (Biotinylated, Anti-Mouse IgG, Anti-Rabbit IgG, Vector Laboratories, Newark, CA, USA code ZB0924, BA-1400).

To reveal horseradish peroxidase (HRP) activity, the glucose oxidase–DAB–nickel method [53] was employed. Following appropriate agitation, the retinas were mounted between two glass slides, while the brain sections were mounted onto gelatinized slides and left to dry at room temperature. After drying, the retinas and brain sections were counterstained with Giemsa and cresyl violet, respectively, followed by dehydration, and were cleared in alcohol and xylene. Coverslips were added with an embedding medium (Entellan).

The specificity of the immunohistochemical patterns was assessed by omitting the primary antibody [54].

### 2.4. Stereological Counting Procedures

- *Microscopy and Optical Fractionator*

In brief, we delineated at all levels in the histological sections the region and layers of the dentate gyrus, digitizing them directly from the sections using a low-power 4.0× objective on an Optiphot 2 microscope (Nikon, Tokyo, Japan) equipped with a motorized stage (MAC200). This system was coupled to a computer running Stereo Investigator software, version 2017.03.3 (MicroBrightField, Williston, VT, USA), used to store and analyze the x, y, and z coordinates of the digitized points to detect and count unambiguously the objects of interest in the dissector probe, and the low-power objective was replaced with a 100× oil immersion plan apochromatic objective (NIKON, NA 1.4) to count the microglia.

- *Area and objects of interest*

The frontiers of the molecular layer of the dentate gyrus are conspicuous; the border between the polymorphic layer and the CA3 region was arbitrarily defined in the horizontal sections with a straight line that connected the tip of the pyramidal cell layer of the CA3 with the two tips of the granular cell layer. The CA3 pyramidal and dentate gyrus granular layers appeared as distinct bands easily distinguished from the polymorphic layer. A straight line was used to distinguish the microglia of the CA3 from the microglia of the polymorphic layer. All the other layers of the dentate gyrus can be distinguished from each other owing to their distinct appearance, and individual microglia were identified on the sections that had been immunoreacted for IBA-1 (See Figure S1).

After selectively immunolabeling the microglia, we quantified the IBA-1-immunolabeled cells in both the control and infected mice using the optical fractionator method [55]. This technique is resistant to histological changes, including tissue shrinkage and damage-induced tissue expansion [56].

For each counting box placed at the molecular layer of the dentate gyrus, the section thickness was meticulously determined using the high-power objective, identifying the upper and lower bounds of the section. Microglial cell bodies that came into focus within the counting box were systematically counted and included in the overall marker sample. This ensures containment within the counting box or at the crossing of the acceptance lines, excluding contact with the rejection line [57].

The total cell numbers were estimated using the number-weighted section thickness to account for uneven cell thickness and distribution at each counting site. The arrangement of counting boxes within a grid was systematically and randomly conducted to achieve an acceptable methodological error coefficient (C.E. < 0.05), utilizing the Scheaffer coefficient, a previously validated and tested parameter [58].

The estimation of the total cell numbers via the optical fractionator involved multiplying the total counted markers within each counting box by three sampling fractions representing the section sampling fraction (ssf), area sampling fraction (asf), and thickness sampling fraction (tsf) after histological procedures. The equation used for estimating the total number of cells is given by:

N = ΣQ × 1/ssf × 1/asf × 1/tsf

where N represents the total number of cells, and ΣQ denotes the number of counted objects (markers) [55,56,59]. Table 1 provides the detailed stereological parameters.

Figure S1 is a composition of screen captures and photomicrographs, with a low-power image capturing the molecular layer of the dentate gyrus (shaded green area) (A), along with a grid utilized for cell counting (B), delineating acceptance (green) and rejection (red) lines within the counting boxes (C). Additionally, (D) presents high-power photomicrography focusing on the IBA-1-immunolabeled microglia, the objects of interest. It is important to note that the counting probes are systematically placed at fixed intervals across the molecular layer of each section, ensuring uniform sampling probability across all regions.

The stereological outcomes from various experimental groups were subjected to parametric statistical analyses utilizing two-tailed *t*-tests. Differences between groups were deemed important at a 95% confidence level (*p* < 0.05).

In the present study, the acceptable level of errors in the cell number estimations was determined by considering the ratio between the intrinsic error introduced by the methodology and the coefficient of variation. To express the accuracy of the estimates, the coefficient of error (CE) was used, and a value of CE ≤ 0 was considered appropriate. This is because the variance introduced by the estimation procedure contributes very little to the observed group variance [58,60]. The experimental parameters for IBA-1 cell marker and region were established in pilot experiments and applied consistently across all animals and regions of interest (Table 2).

### 2.5. Three-Dimensional Reconstructions

When an organism experiences a perturbation to brain homeostasis, its microglia morphology often changes in an attempt to restore homeostasis and prevent tissue damage [61,62]. It is believed that form and function are interconnected, and the morphology of microglia can be examined to determine their contribution to homeostasis and imbalance. Multiple reactive microglial states are selected through the discrimination of discrete perturbations within the brain parenchyma in both physiological and pathological conditions [63]. This substantial variability is reflected in the dispersion profiles in the morphometric analyses of hundreds of cells reconstructed in each experimental group in the present work, requiring multivariate analysis for their morphological classification.

Microscopic three-dimensional reconstruction techniques, coupled with hierarchical cluster analysis of morphometric features, have been pivotal in classifying microglia across diverse species, elucidating their states under both homeostatic and non-homeostatic conditions [37,50,64,65]. This methodology provides an objective framework for characterizing and quantifying the observed morphological changes, offering insights into microglial alterations during normal homeostasis and neuropathological states [66,67,68].

### 2.6. Stereological Sampling Approach and Hierarchical Cluster Analysis to Classify Microglia

Here, we used hierarchical cluster and discriminant analyses to classify microglial morphology. Microglia in the molecular layer of the dentate gyrus were carefully examined using a 100× oil immersion plan fluoride objective at high resolution (NIKON, numerical aperture—NA 1.3, depth of field—DF = 0.19 µm).

We captured and processed the images using Neurolucida neuron tracing software (Neurolucida Explorer 11.03; M.B.F. Bioscience, Williston, VT, USA). To create the three-dimensional (3D) reconstructions, images were acquired with a high-resolution video camera and displayed on HD monitors. The morphological intricacies of the microglia were carefully digitized point by point using Neurolucida and 3D reconstructions exclusively performed on cells displaying unequivocally intact arbors, with exclusion criteria applied to cells possessing artificially damaged branches (either at the surface or base of the sections) or incomplete immunolabeling. Terminal branches, usually finer in structure, were systematically confirmed before cell selection for 3D reconstruction. Microglia were selectively labeled with the IBA-1 antibody, and the photomicrographs depicted various magnifications of microglia from the 6-month-old mice in the molecular layer of the dentate gyrus (Mol-DG). To address *z*-axis shrinkage, we applied a correction factor of 75%.

We used systematic random sampling to ensure an equal probability of selecting all the dentate gyrus regions in the sample. Random and systematic horizontal section samples were taken from sections covering the entire hippocampal formation. The Mol-DG boxed areas were marked to indicate the location of individual microglia that were selected for 3D reconstruction. Figure S1D provides a detailed, high-powered view of the IBA-1 immunolabeled microglia.

To assess the morphological features in the uninfected (control) and *T. gondii*-infected mice, we employed multivariate hierarchical cluster analysis, a methodology previously applied to classify neuronal types in the nucleus of the solitary tract [69], astrocytes in the dentate gyrus [70], and microglia in the hypoglossal nucleus [68]. We used morphometric features with multimodality indices (MMIs) above 0.55 and applied Ward’s hierarchical clustering method, as suggested elsewhere [69]. We estimated the MMI based on the skewness and kurtosis of our sample for each morphometric variable as MMI = MMI = [M3^2^ + 1]/[M4 + 3 (n − 1)^2^/(n − 2) (n − 3)], where M3 is skewness, M4 is kurtosis, and n is the sample size [69,71].

Some morphological features showed multimodality indices (MMIs) greater than 0.55. This indicates that their distributions were potentially multimodal or at least bimodal. The morphological classification was explained using a dendrogram and Ward’s method with standardized variables. Discriminant function analysis was then performed to determine the primary variables contributing to cluster formation. This analysis aimed to determine whether the clusters of cell morphologies significantly differed in the mean value of a particular variable. Based on that variable, group membership could be predicted.

We used multivariate F-tests to compare matrices of total variance and covariance to identify any important differences between groups of variables with MMIs exceeding 0.55. This procedure helped us to identify the morphometric variables that best differentiated the suggested microglia classes identified by cluster analysis. We also computed arithmetic means and standard deviations for the selected variables that proved to be the most reliable predictors for the microglia groups.

We conducted t-tests and two-way ANOVA to find microglia cluster differences within groups. We aimed to detect morphological variances in the microglia morphometric features from the dentate gyrus of the control and infected mice. We selected a representative cell for each group from three dentate gyrus cell types from the control and infected mice. We used a distance matrix to identify the cell closest to all others, indicating its representativeness. This involved calculating a weighted scalar Euclidean distance between all the cell pairs within a group, using the STATISTICA data analysis software, version 12 (StatSoft, Inc. Palo Alto, CA, USA, (2014)), for the Euclidean distance matrices and sum of distances.

For behavior changes, we used ANY-maze, Biostat 5.4, and GraphPad Prism 9 for the statistical analysis, removing outliers based on standard deviation. *t*-tests were used for two related samples in the open field test results, and two-way ANOVA was used to assess the interactions between the experimental conditions and disease progression over time.

## 3. Results

### 3.1. Comparative Microglial Numerical Changes in Control and T. gondii-Infected Mice

Comprehensive analysis of the microglial counts in both control and infected albino Swiss mice, presented in Table 1, reveals that ocular infection with *T. gondii* at 12 days post-infection (dpi) significantly increases the number of microglial cells within the molecular layer of the dentate gyrus. In contrast, BALB/c mice infected with the same pathogen also exhibited changes in their microglial numbers, albeit to a lesser extent, which peaked at a later time point (22 dpi).

The infected albino Swiss mice showed an important 1.6-fold increase in their total microglia compared to the control mice. This increase indicates strong microgliosis induction in the molecular layer of dentate gyrus 12 days post-infection. The control mice had an estimated total of 4862 ± 932 microglia, while the infected mice had an estimated total of 7959 ± 917 microglia. The two-tailed *t*-test showed t = −5.54, *p* = 0.0004, and Cohen’s effect size D = 3.35, considered an important and large effect. Cohen’s effect size for BALB/c was also significant (>0.8). The control mice had an estimated total of 4349 ± 1171 microglia, while the infected mice had an estimated total of 5870 ± 531 microglia, with t = −2.6, *p* = 0.03, and D = 1.8. However, the effect size for BALB/c was approximately half of what was observed in albino Swiss mice. For a more detailed analysis of the numerical changes in the microglia within the BALB/c lineage, please refer to the study published elsewhere [49].

### 3.2. Behavioral Changes

1. Mouse preference in the three-chamber olfactory test and in the open arena

At the acute stage of the disease, all the infected Swiss albino mice exhibited classic sickness behaviors, including ruffled fur, reduced locomotor activity, tremors, and a hunched posture, though the severity varied. Of the 21 Swiss albino mice, 5 succumbed to the infection on the 11th day post-infection. The surviving 16 mice underwent behavioral testing and were euthanized on the 12th day post-infection for neuropathological analysis. In contrast, the BALB/c mice (n = 27) infected with the same pathogen did not show typical acute sickness behaviors but did exhibit increased immobility.

**Figure 1:**
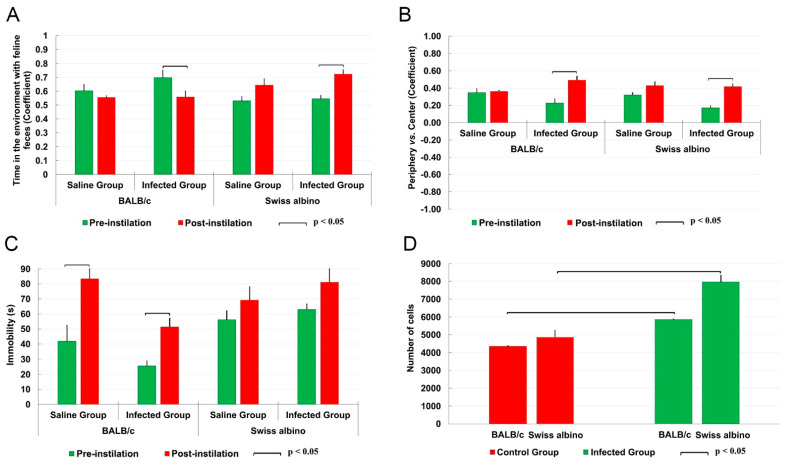
Comparative behavioral changes (**A**–**C**) and stereological estimation of total microglial numbers in the molecular layer of the dentate gyrus (**D**) in infected (green columns) and control (red columns) mice. Olfactory findings in infected mice (**A**) revealed an important increase (BALB/c) and reduction (Swiss albino) in time spent in compartments with feline odorants compared to controls. In the open field arena, infected BALB/c mice displayed an important increase in immobility compared to controls, while Swiss albino mice maintained their mobility levels (**B**); both strains showed consistent distribution patterns between the center and periphery of the open field (**C**). Notably, infected mice from both strains exhibited an important increase in the total number of microglia (**D**).

In the olfactory test, the BALB/c mice spent more time in the compartment containing feline feces, in contrast to the albino Swiss mice, who exhibited a reduced time or an equivalent time in this environment, as shown in Figure 1A. This suggests the presence of differing responses to feline odors between the two strains. Both strains exhibited similar tendencies in the open field test, with a preference for staying within the peripheral space rather than venturing into the center (Figure 1B). Regarding locomotor activity, the infected BALB/c mice showed an increase in immobility when compared to their uninfected counterparts, whereas no significant difference in immobility time was observed between the infected and uninfected albino Swiss mice (Figure 1C).

These observations of behavioral changes, both similar and contrasting, among the strains were accompanied by an important increase in the number of microglia in the molecular layer of the dentate gyrus, as illustrated in Figure 1D.

### 3.3. Microglial Morphotypes in T. gondii-Infected and Control Mice

**Figure 2:**
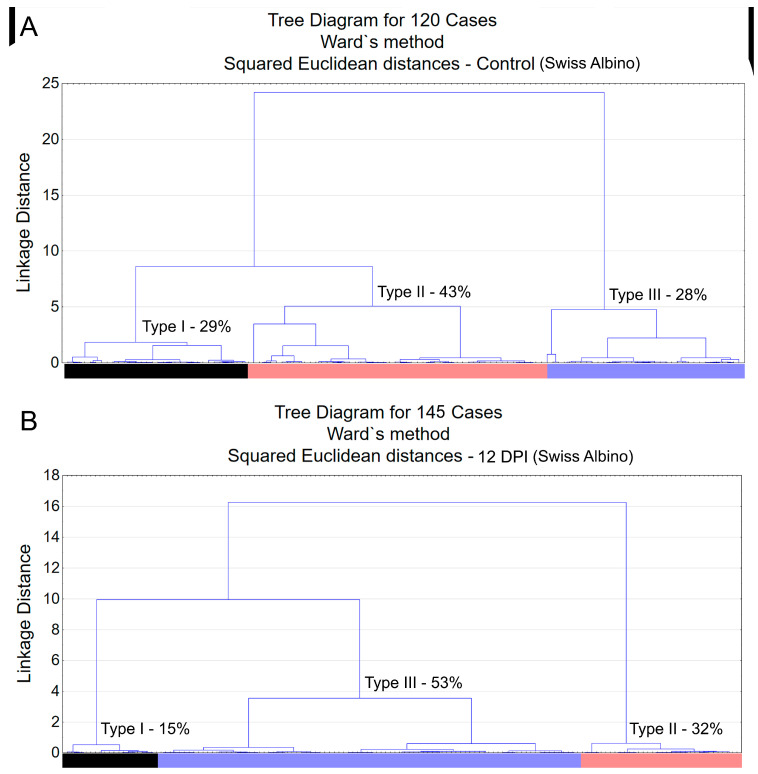
Dendrogram resulting from hierarchical cluster analysis applied to multimodal variables extracted from 3D reconstructions of microglia in the molecular layer of the dentate gyrus of control animals (**A**) and *T. gondii*-infected (**B**) Swiss albino mice. Three distinct morphological families are observed in both control and infected groups, with notable shifts in the percentage values of morphotype III following infection. Distinct colors identify different morphological families of microglia, which are reproduced in the graphic representation of microglial discriminant analysis.

Morphological analysis of immunostained microglia revealed three distinct types of microglia, type I, type II, and type III, as shown in Figure 2. These types represent varying morphological families of microglia, differing in their convex hull volume and complexity. In the Swiss albino mice infected with *T. gondii*, there was a notable decrease in both convex hull volume and the morphological complexity of the microglia compared to the controls. This reduction was key to understanding the cluster formation within the experimental groups. A pivotal observation was the altered distribution of microglia types in the infected mice, with an increase in type III and a decrease in types I and II, suggesting changes in microglial reactivity indicative of neuroinflammation.

**Figure 3:**
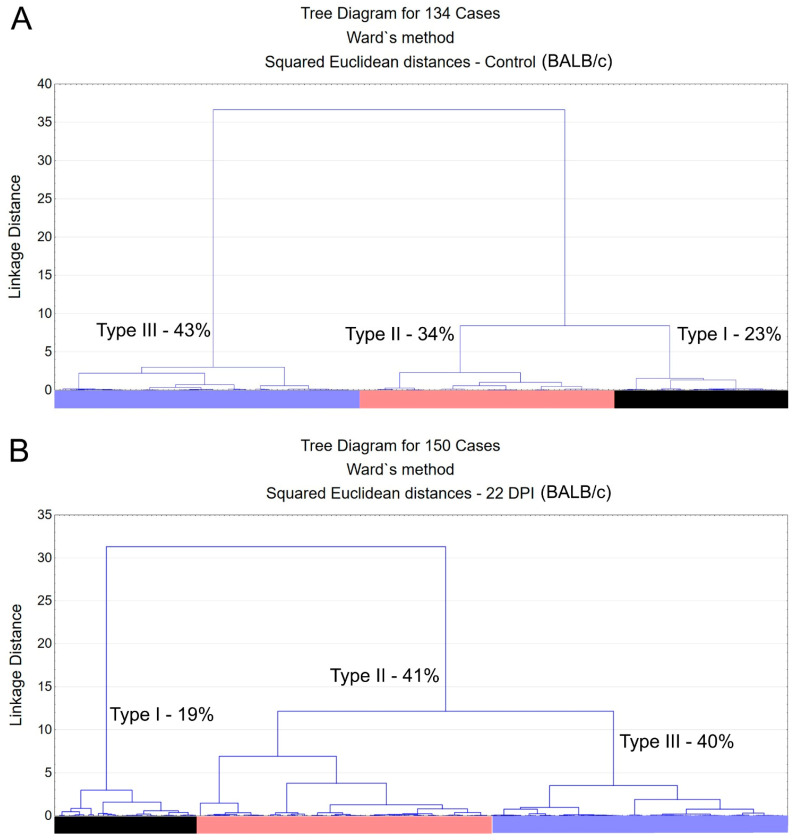
Dendrogram resulting from hierarchical cluster analysis applied to multimodal variables extracted from 3D reconstructions of microglia in the molecular layer of the dentate gyrus of control animals (**A)** and *T. gondii*-infected (**B**) BALB/c mice. Subtle alterations in relative percentage shifts of morphological types are observed following ocular infection by *T. gondii* in the BALB/c lineage. Distinct colors identify different morphological families, which are consistently represented in the graphic representation of discriminant analysis.

**Figure 4:**
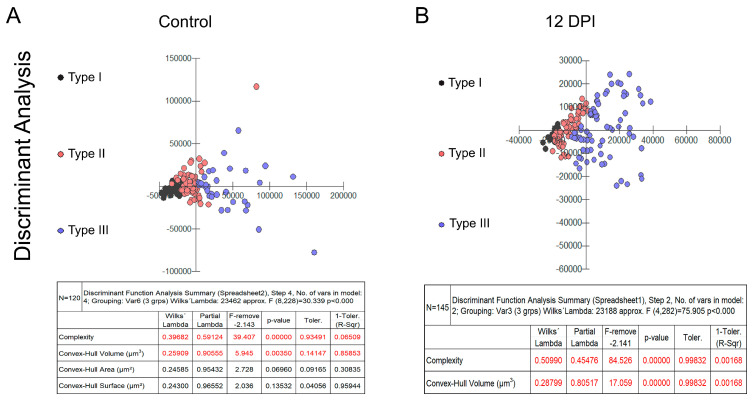
Graphic representations of discriminant analysis and corresponding tables displaying the relative contributions of morphometric variables influencing cluster formation in control (**A**) and infected (**B**) groups within the Swiss albino lineage. Note the distinctive shift in the relative proportion of type III microglia (depicted as blue dots), along with the proportional decrease in types I (black dots) and II (red dots) among the infected subjects.

**Figure 5:**
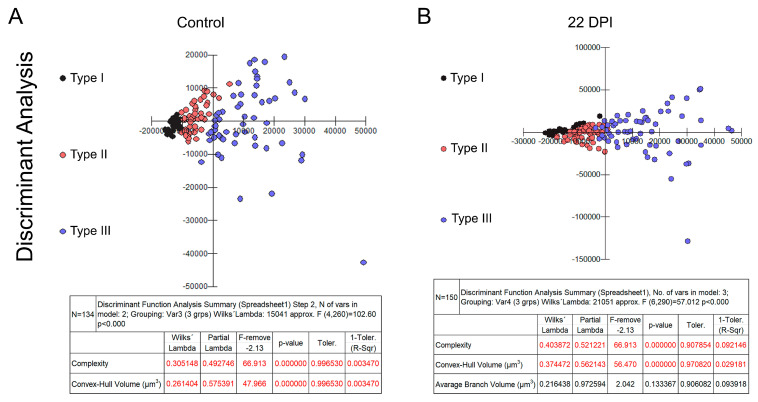
Graphic representations of discriminant analysis and corresponding tables displaying the relative contributions of morphometric variables influencing cluster formation in control (**A**) and infected (**B**) groups within the Swiss albino lineage. Note the distinctive shift in the relative proportion of type III microglia (depicted as blue dots), along with the proportional decrease in types I (black dots) and II (red dots) among the infected subjects.

Figure 2 and Figure 3 present dendrograms from our hierarchical cluster analysis, illustrating the three distinct morphological microglia families in both the control and infected groups of the albino Swiss and BALB/c strains. Further, Figure 4 and Figure 5 depict the use discriminant function analysis to validate the cluster analysis, highlighting the influence of each morphometric feature on the cluster differentiation.

Type I, II, and III microglia exhibit progressive changes in their morphological complexity and convex hull volume. Our findings highlight a shift towards increased type III microglia in the infected mice, indicating a change in microglial population dynamics and suggesting an adaptation to infection.

**Figure 6:**
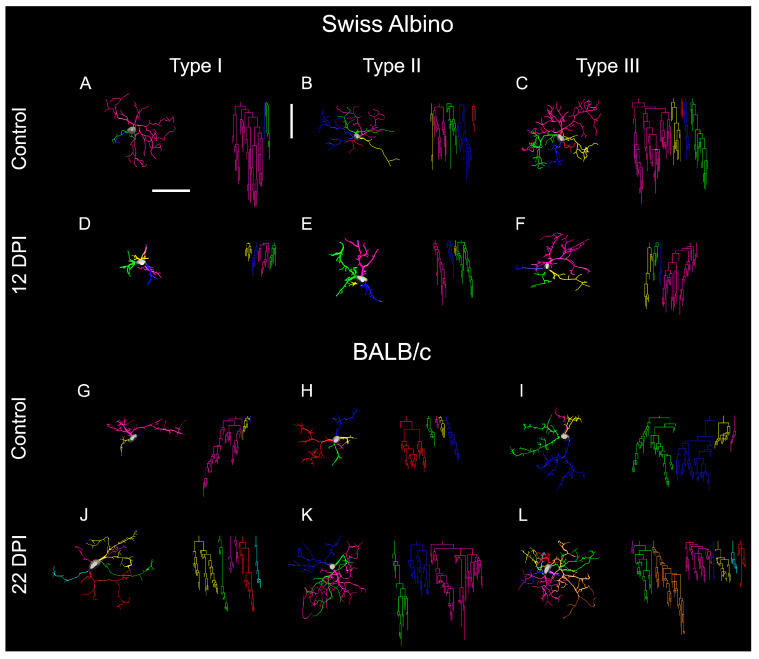
Microglial morphological alterations induced by *T. gondii* ocular infection in the molecular layer of the dentate gyrus of BALB/c and Swiss albino mice. Microglia from control (**A**–**C**,**G**–**I**) and from infected (**D**–**F**,**J**–**L**) display contrasting morphological changes both in Swiss albino (**A**–**F**) and BALB/c (**G**–**L**) mouse models. Each row exhibits cells representing the average of type I, II, and III categories, illustrating a spectrum of progressive morphological complexity. These categories denote variations in morphological features, particularly greater convex hull volume and morphological complexity, which were identified as key contributing variables to cluster formation within each experimental group. Notably, both strains displayed important yet opposed microglial morphological changes: BALB/c mice exhibited extended microglial branches by 22 dpi, whereas Swiss albino counterparts showed shortened branches by 12 dpi. Horizontal and vertical scale bars: 25 µm.

Figure 6 offers a visual summary of the microglial alterations following ocular *T. gondii* infection in both BALB/c and Swiss albino mice. It compares the representative microglia of each morphotype, underscoring the differences in coverage, branch thickness, and complexity. The infected BALB/c mice displayed expanded microglial arbors with greater complexity, whereas the Swiss albino mice showed reductions in these aspects, illustrating the diverse impacts of infection on microglial morphology across strains.

**Figure 7:**
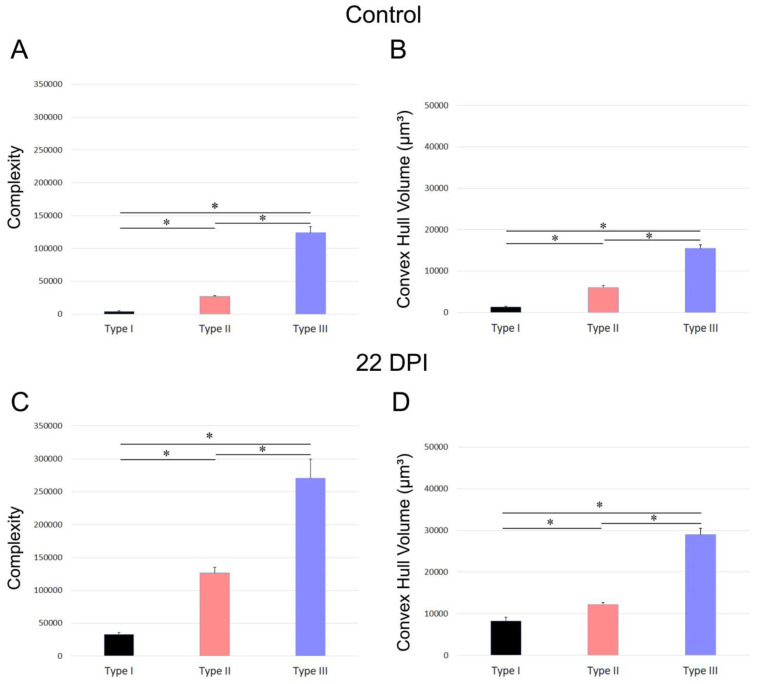
Graphic representation of mean values and standard errors of the morphometric features of microglia of the molecular layer of dentate gyrus of BALB/c strain that most contributed to cluster formation. (**A**,**B**) indicate mean values and standard deviation for microglia of control mice and (**C**,**D**) represent mean ± s.d. for microglia of infected mice at 22 DPI. Notice that the three distinct morphotypes were affected differentially by *T. gondii* infection in BALB/c. * Significant difference *p* < 0.05.

**Figure 8:**
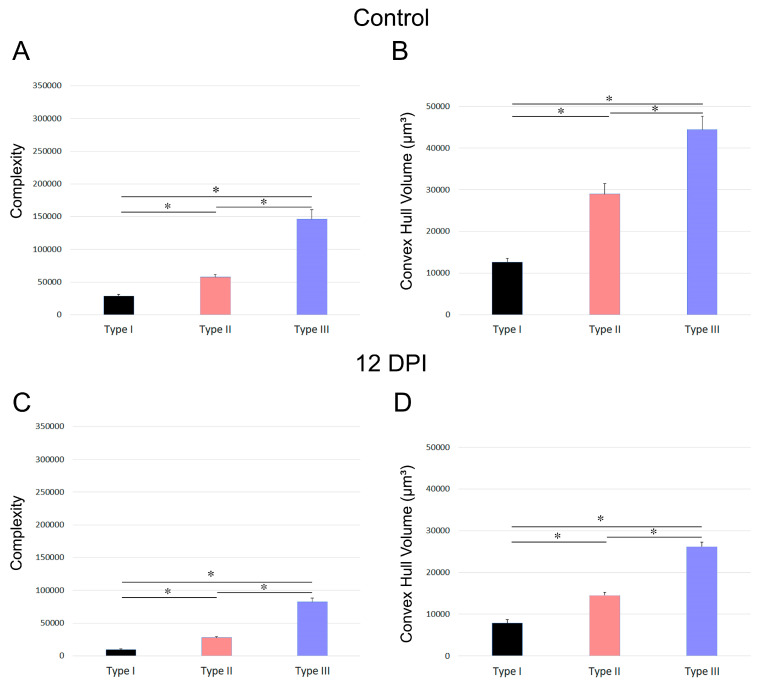
Mean values and standard errors of morphometric features of microglia within the molecular layer of dentate gyrus of Swiss albino strain, significantly contributing to cluster formation. (**A**,**B**) indicate mean values and standard deviation for microglia of control mice and (**C**, **D**) represent mean ± s.d. for microglia of infected mice at 12 DPI Notably, the differential impact of *T. gondii* infection on the three distinct morphotypes is also evident within the Swiss albino strain. * Significant difference *p* < 0.05.

Figure 7 and Figure 8 depict the differences in convex hull volume and morphological complexity across the three identified microglia morphotypes in both the control and infected mice of the BALB/c and Swiss albino strains. Figure 7 presents the mean values and standard errors for key morphometric features of the microglia in the dentate gyrus molecular layer, crucial for cluster formation. It highlights the variable impact of *T. gondii* infection on the morphotypes in BALB/c mice. Conversely, Figure 8 focuses on the mean values and standard errors of important morphometric characteristics of the microglia in the Swiss albino strain, illustrating the distinct responses of microglia morphotypes to infection.

The analysis reveals contrasting microglial responses between the BALB/c and Swiss albino strains. In the BALB/c mice, the microglia displayed expanded branches, while the Swiss albino mice exhibited a pattern of branch retraction. Figure 7 and Figure 8 underscore these differences by showing the average convex hull volume occupied by the microglia, indicating expansion in the BALB/c lineage and reduction in the Swiss albino lineage.

**Figure 9:**
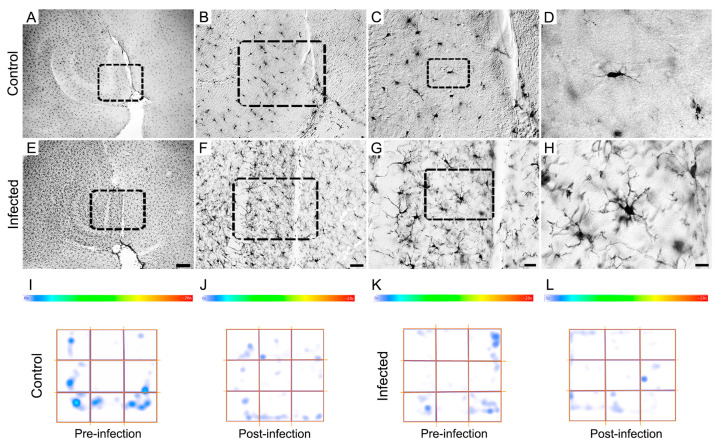
Photomicrographs at 12 days post-infection (dpi) of microglia in the molecular layer of the dentate gyrus of Swiss albino brain sections captured with low-, medium-, and high-power objectives from control (**A**–**D**) and *T. gondii*-infected (**E**–**H**) subjects, accompanied by corresponding heat maps depicting immobile time (**I**–**L**). Behavioral data were collected at 12 dpi from control and infected subjects showing no apparent sickness behavior. Microglial photomicrographs were obtained from the molecular layer of the dentate gyrus of the same animals. Interestingly, despite displaying reactive microglial morphology, infected animals did not exhibit a significant increase in immobility following *T. gondii* infection. Scale bars: (**A**,**E**): 200 µm; (**B**,**F**): 50 µm; (**C**,**G**): 20 µm; and (**D**,**H**): 10 µm.

The microglia in the molecular layer of the dentate gyrus of the Swiss albino mice infected with *T. gondii* exhibit a unique reactive morphology compared to the controls, as summarized in Figure 9A–H. This reactivity, evident across all the distinguished morphotypes, correlates with behavioral changes, such as increased avoidance of feline odors (Figure 1A) and more time spent in the peripheral zones of the open arena (Figure 1B). However, no important difference was found in immobility between the control and infected Swiss albino mice at 12 dpi.

Figure 9I–L shows a heat map of the immobility time in the open arena based on photomicrographs of an animal whose immobility pattern closely matches the average, illustrating this behavior.

The correlation between microglial reactivity, behavioral alterations, and the progression of toxoplasmosis in BALB/c and Swiss albino mice suggests that differences in the regulation of the inflammatory response may influence the susceptibility and resilience of a strain to infection.

## 4. Discussion

This study investigated sickness behavior and the microglial response as indicators of the inflammatory response to toxoplasmosis in two mouse strains: BALB/c and Swiss albino. Notably, owing to the need to sacrifice the Swiss albino mice at 12 days post-infection (dpi), we were unable to evaluate the chronic-phase outcomes in this lineage. For the comparison, we utilized raw data from previous BALB/c lineage behavioral tests [49] with the available data from the Swiss albino lineage. The BALB/c lineage exhibited lower sensitivity to the RH strain, surviving the acute phase and progressing to the chronic phase with cyst formation in the retina and brain. Chronic-phase data were collected at 42 days post-infection [49].

Our findings showed that the BALB/c mice, during the chronic phase, displayed increased time spent in the feline-scented compartment, contrasting with the Swiss albino mice, who showed decreased time spent in this compartment during the acute phase. Based on these findings, we suggest that the increase in time spent in the feline-scented compartment is specific to the chronic phase when parasites are encysted. The findings reveal differences in microglial response and disease progression between these strains, suggesting that BALB/c and Swiss albino mice regulate the inflammatory response to infection in distinct ways. This differential regulation may account for the observed variations in the tolerance and resistance of each strain to the parasite.

The host immune system mounts a coordinated response to infections by pathogens, with the primary goal of restoring homeostasis and safeguarding the individual from tissue damage. To counteract the pathogen, the host employs two main strategies: resistance and tolerance [6,7,72]. The specific nature of these responses can differ across species and even among individuals within the same species. Resistance encompasses both innate and adaptive immune responses designed to curb pathogen replication. In contrast, disease tolerance involves the immune system permitting the coexistence of the pathogen within the host, often with minimal or no clinical signs, while still allowing sufficient pathogen replication for transmission.

The behavioral observations in this study indicate that during the acute phase of infection, Swiss albino mice exhibited a dysregulated immune response, leading to the death of five mice by 11 dpi. The mice that survived exhibited reduced aversion to feline odors at 12 dpi and spent more time at the periphery of the open field but did not demonstrate an important increase in periods of immobility. In contrast, all the infected BALB/c mice survived the initial phase of the infection. By 22 dpi, the BALB/c mice exhibited decreased avoidance of feline odors, longer durations of immobility, and increased time spent at the open field’s edges. By 43 dpi, the BALB/c mice continued to spend more time in areas with feline odors, suggesting that the behavioral alterations noted at 22 dpi may have become enduring. The immune response of the infected Swiss albino mice was similar to that observed in C57Bl6 infected mice [73,74], and previous reports comparing the BALB/c and C57Bl6 lineages have also reported an increase in macrophage numbers during the late stages (56 dpi) of the disease in BALB/c mice compared to C57Bl6 infected mice. Additionally, activation pathways associated with neuropathology and neuroinflammation in BALB/c mice are downregulated compared to their counterparts in C57BL/6 mice [73,74,75]. Such studies highlight how alterations in the host immune response influence pathogenicity to *T. gondii* infection.

Between 22 and 43 days post-infection, the BALB/c mice displayed behavioral changes that correlated with increased microglial activity. The microglia showed expanded trees and greater morphological complexity. By 43 dpi, however, the microglial response had decreased, and cysts were observed in the retina and cerebral cortex. Although the three microglia morphotypes identified at 22 and 43 dpi persisted, they exhibited morphometric features similar to those of the control mice, as shown in Figure S2. Further analysis of microglial arbor volume and shape showed that the BALB/c mice had more complex and voluminous microglia, while the Swiss albino mice displayed shrunken and rounded microglia, similar to macrophages. These observations suggest different phases of the microglial inflammatory response between the strains, with differing functions. In the Swiss albino mice, a surge in microglial activity at 12 dpi coincided with severe sickness behavior and fatalities at 11 dpi. Conversely, the BALB/c mice exhibited behavioral and microglial changes peaking at 22 dpi, which returned to baseline with brain cyst presence at 43 dpi. It might be speculated that the delayed increased microglia number might have protected the BALB/c mice from toxoplasmic encephalitis. In contrast, the Swiss albino mice exhibited a doubling in microglia number with macrophage-like morphologies at 12 days, indicating a potentially dysregulated inflammatory response that led to more severe outcomes in this strain and the downstream failure to develop an odor response. Here, myeloid cell recruitment from the bloodstream into the *T. gondii*-infected brain may have contributed to the fatal toxoplasmic encephalitis in the albino Swiss mice, as previously described in C57Bl/6J see [76]. T cells, particularly CD8+ T cells, also exhibited a marked increase in number in infected C57Bl/6J mice, concomitant with heightened levels of macrophages and activated microglia up to 28 dpi, after which they stabilized. Nonetheless, the virulence and persistence of the parasite in mice are tied to the host’s genetic background.

The severity of toxoplasma infection in mice is determined by the ability of the parasite to counteract the host’s resistance to infection, which appears to be targeted at interferon-gamma (IFN-γ) through its parasite kinase [77]. During the acute infection stage, there is an increase in neuronal cell death, the activation and infiltration of microglia, the expression of both inflammatory and anti-inflammatory cytokines, and Stat1 phosphorylation, as well as enhanced microglia reactivity and associated gene transcripts [78]. This suggests that the failure to evade the activation of innate and cellular inflammatory responses, accompanied by downstream neurodegeneration, might be responsible for an excessive inflammatory response [78]. Microglia and macrophages are known to produce IFN-γ in the brain following infection with *T. gondii*, which is responsible for controlling the behavior of tachyzoites through chemokine and MHC antigen expression [12,79]. This mechanism may stop parasite proliferation in the early stages of the disease in BALB/c mice but not in Swiss albino mice [12]. It has also been shown that the host’s tolerance mechanisms require the parasite’s engagement of the scavenger receptor CD36. This engagement is critical for the re-establishment of tissue homeostasis and survival following the acute phase of infection [77].

It was demonstrated that the behavior of rodents infected with *T. gondii* is affected by the parasite strain and the immune response of the mouse model lineage [80,81]. Variations among the three primary strains of *T. gondii* and their specific interactions within the infected host significantly influence the severity of infection [82]. The acute virulence of the RH strain, which was used in this study, is consistent with elevated tissue burdens compared to the non-virulent type II and type III lineages [83]. In a study using BALB/c mice, researchers investigated the impact of using different strains—ME-49 (type II) and VEG (type III)—of *T. gondii* on the outcome [80]. The investigation encompassed assessments of learning and memory capabilities, locomotor activity, and aversion to feline odor. Additionally, the study measured the humoral immune response, the levels of total IgG anti-toxoplasma, and the parasite load within the CNS. The findings revealed that mice infected with the VEG strain exhibited several notable differences compared to those infected with the ME-49 strain. These included increased levels of total IgG anti-toxoplasma, a higher parasite burden in the CNS, impaired long-term memory, reduced mobility, and a diminished aversion to feline odor. This suggests that the genetic variation between the *T. gondii* strains significantly influences the infection’s impact on the host, affecting both the immune response and behavioral outcomes [84].

Comprehensive transcriptional profiling of mice during both the acute and chronic phases revealed that a distinct subset of host genes is induced in the immune response, and some are notably more pronounced during the chronic stage [85]. This finding indicates an enduring interplay between the pathogen and host that remains evident in the late stages when brain cysts are detected in the parenchyma [85]. In the early stages of a systemic infection caused by *T. gondii*, the parasite must express a different set of surface antigens inside a modified parasitophorous vacuole to encyst [75,86]. According to studies conducted on C57BL/6 mice, by 28 dpi, a chronic infection was established, with a late-stage bradyzoite phenotype dominating the infection. Transcriptomic analysis of C57BL/6 and BALB/c mice during progressive chronic *T. gondii* infection showed important changes in host genes [75]. It is of interest to note that during *T. gondii* infection, human neutrophil-like cells have been found to demonstrate antimicrobial responses to chronic cysts, suggesting their participation in clearing the parasite [87,88].

- *Technical limitations*

Comparative studies often yield conflicting results, influenced by factors such as divergent animal lineages, variations in histological techniques, and inconsistencies in defining specific objects and areas for stereological estimates and three-dimensional (3D) reconstructions. To mitigate these sources of non-biological variation, we standardized the tissue processing across all samples. It is important to note that stereological estimates and 3D reconstructions are sensitive to mechanical factors, with severe shrinkage along the *z*-axis potentially manifesting as curled branches. To counteract this, we took samples from the central portion of the *z*-axis. Additionally, we adjusted all the microglial reconstructions for *z*-axis shrinkage, adhering to the guidelines recommended by Carlo and Stevens [89]. To ensure the reliability of our observations, different researchers independently reconstructed microglia within the same regions, allowing us to identify and consider any variations in our data.

## 5. Conclusions

In this study, we investigated the alterations in the microglia within the molecular layer of the dentate gyrus and examined the behavioral responses of two mouse strains, BALB/c and Swiss albino, to ocular toxoplasmosis infection. Our results, in conjunction with findings from other researchers, underscore the pivotal role of inflammation in influencing the severity of toxoplasmosis outcomes. Noteworthy is our observation of distinct differences in disease progression between the two mouse strains, highlighting the central role of the inflammatory response in determining an infected host’s capacity to either tolerate or combat the infection. Specifically, the findings point towards a compromised inflammatory response in Swiss albino mice, suggesting that the efficacy of tolerating or resisting *Toxoplasma gondii* infection is intricately linked to how inflammation is sensed. This research sheds light on the intricate relationship between microglial activation, the inflammatory process, and the host’s defense mechanisms against pathogenic invasions.

## Figures and Tables

**Table 1:** Stereological estimates of microglia total number in the molecular layer of the dentate gyrus of control and *T. gondii*-infected Swiss albino female mice.

Animal	Total Number	Thickness (µm)	Scheaffer CE
(Uninfected Swiss albino)			
Animal 6	4448	41.20	0.042
Animal 7	4243	40.50	0.043
Animal 12M	6018	34.80	0.046
Animal 6M	5688	35.20	0.048
Animal 7M	3913	22.50	0.043
Mean	4862	34.84	0.043
SD	931.9	7.498	0.044
CV	0.191	0.215	
CV²	0.036	0.046	
CE^2^	0.001	0.001	
CE² / CV²	0.027	0.021	
CV²—CE²	0.035	0.045	
CVB² (%)	97.22	97.82	
(12 DPI Infected Swiss albino)			
Animal 18	6392	34.80	0.032
Animal 22	7355	41.00	0.033
Animal 37	8793	51.50	0.033
Animal 41	8640	37.40	0.029
Animal 42	8347	38.10	0.029
Animal 43	8229	51.00	0.037
Mean	7959	42.30	0.032
SD	916.8		
CV	0.115	0.170	
CV^2^	0.013	0.020	
CE^2^	0.001	0.001	
CE^2^/CV^2^	0.076	0.050	
CV^2^−CE^2^	0.012	0.019	
CVB^2^ (%)	92.30	95.00	

CVB^2^ = CV^2^ − CE^2^; CV = coefficient of variation; CE = coefficient of error; CVB = biological coefficient of variation; SD = standard deviation.

**Table 2:** Stereological parameters for microglia counting in the molecular layer of the dentate gyrus of Swiss albino female mice.

Animal	Thickness (µm)	No. of Sections	No. of Probes	Probe (µm)	Grid (µm)	Dissector Height (µm)	Interval
Control							
Animal 6	41.2	6	721	50 × 50	70 × 70	15	1/3
Animal 7	40.5	6	706	50 × 50	70 × 70	15	1/3
Animal 12M	34.8	5	492	50 × 50	70 × 70	15	1/5
Animal 6M	35.2	5	438	50 × 50	70 × 70	15	1/5
Animal 7M	22.5	5	420	50 × 50	70 × 70	15	1/5
12 DPI							
Animal 18	34.8	5	638	50 × 50	70 × 70	15	1/3
Animal 22	41.0	6	810	50 × 50	70 × 70	15	1/3
Animal 37	51.5	6	797	50 × 50	70 × 70	15	1/3
Animal 41	37.4	6	907	50 × 50	70 × 70	15	1/3
Animal 42	38.1	5	808	50 × 50	70 × 70	15	1/3
Animal 43	51.0	6	661	50 × 50	70 × 70	15	1/3

## Data Availability

The data presented in this study are available on request from the corresponding author.

## Supplementary Materials

The following supporting information can be downloaded at https://www.mdpi.com/article/10.3390/biomedicines12071420/s1. Figure S1. Stereological sampling: Low-power photomicrographs (A,B) displaying the molecular layer of the dentate gyrus, the area of interest, alongside the sampling grid (C). Additionally, a high-power photomicrograph (D) showcases IBA-1-immunolabeled microglia (the object of interest). Scale bar: A: 250 µm; D: 25 µm. Figure S2. The recovery of BALB/c microglia in the molecular layer of dentate gyrus was observed 43 days after infection. At this point, only minor morphological changes were observed, and all the morphological changes induced by *T. gondii* infection at 22 dpi had disappeared. The analysis methods used included hierarchical cluster analysis (A), discriminant function analysis (C,E), morphological complexity (B), and convex hull volume (D).
