# Supplementary material for: Contrasting Disease Progression, Microglia Reactivity, Tolerance, and Resistance to Toxoplasma gondii Infection in Two Mouse Strains
Source: Biomedicines. 2024 Jun 26;12(7):1420. doi: 10.3390/biomedicines12071420 (PMC11274029; doi:10.3390/biomedicines12071420)
Supplement: Supplementary file 1 [file biomedicines-12-01420-s001.zip › Supplementary Figure S1 .pdf]

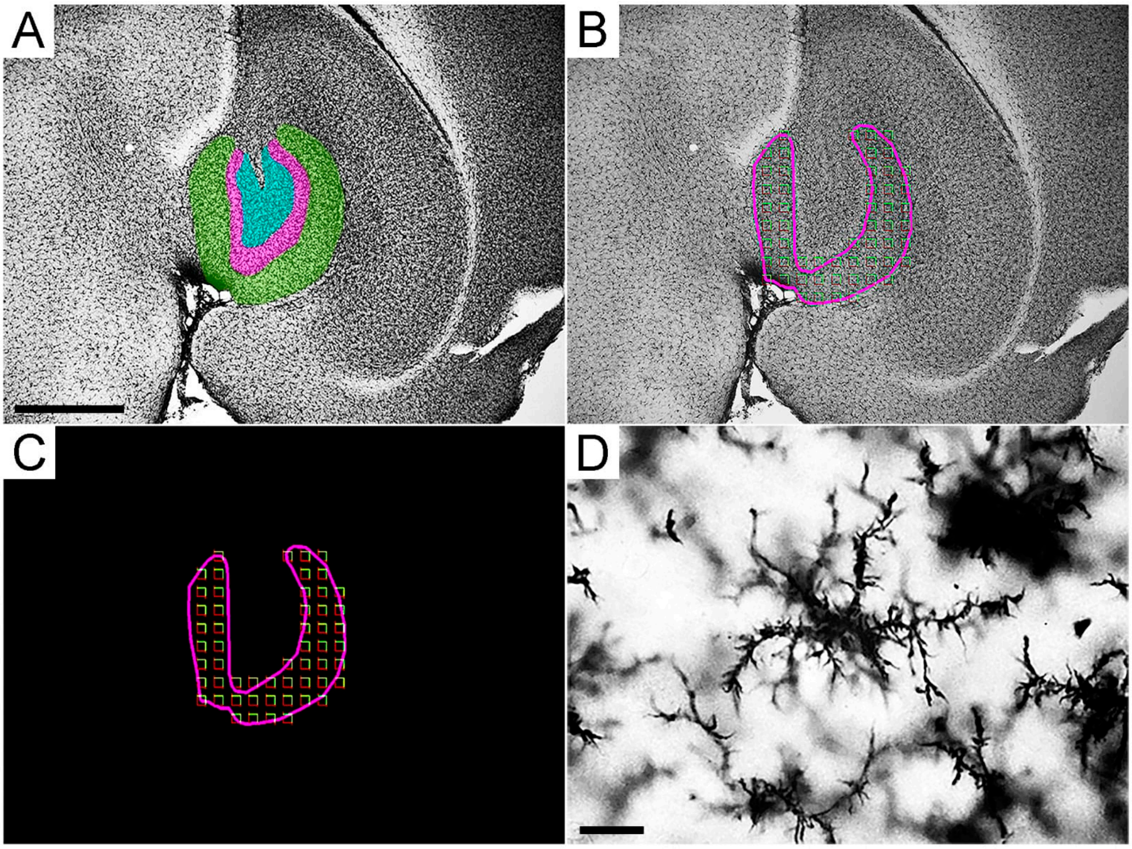

Figure S1. Stereological sampling: Low-power photomicrographs (A,B) displaying the molecular layer of the dentate gyrus, the area of interest, alongside the sampling grid (C). Additionally, a high-power photomicrograph (D) showcases IBA-1-immunolabeled microglia (the object of interest). Scale bar: A: 250 µm; D: 25 µm.
