# Supplementary material for: Contrasting Disease Progression, Microglia Reactivity, Tolerance, and Resistance to Toxoplasma gondii Infection in Two Mouse Strains
Source: Biomedicines. 2024 Jun 26;12(7):1420. doi: 10.3390/biomedicines12071420 (PMC11274029; doi:10.3390/biomedicines12071420)
Supplement: Supplementary file 1 [file biomedicines-12-01420-s001.zip › Supplementary Figure S2.pdf]

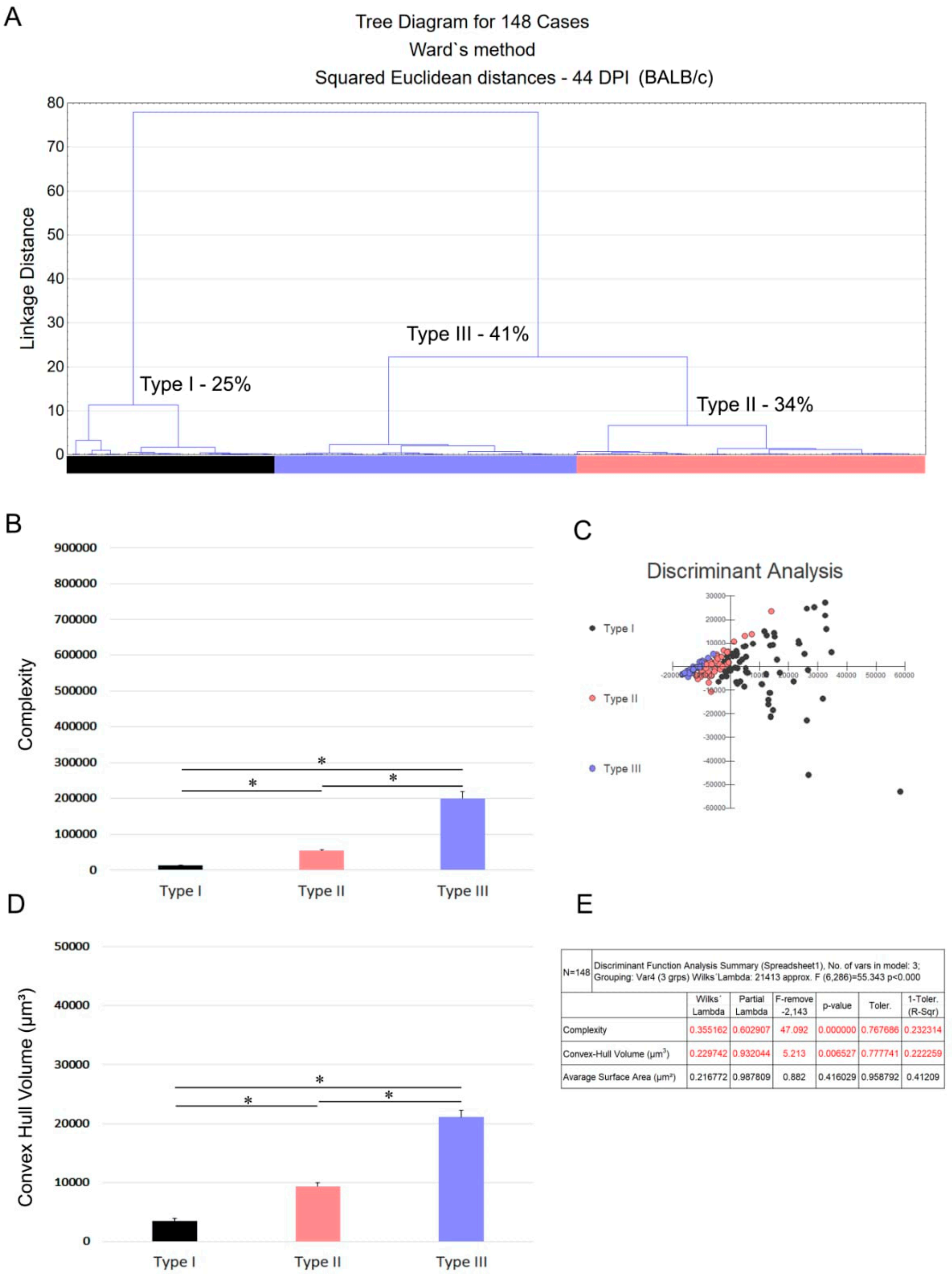

N=148	Discriminant Function Analysis Summary (Spreadsheet1), No. of vars in model: 3; Grouping: Var4 (3 grps) Wilks`Lambda: 21413 approx. F (6,286)=55.343 p<0.000					
	Wilks` Lambda	Partial Lambda	F-remove -2,143	p-value	Toler.	1-Toler. (R-Sqr)
Complexity	0.355162	0.602907	47.092	0.000000	0.767686	0.232314
Convex-Hull Volume (μm^3)	0.229742	0.932044	5.213	0.006527	0.777741	0.222259
Avarage Surface Area (μm^2)	0.216772	0.987809	0.882	0.416029	0.958792	0.41209

Figure S2. The recovery of BALB/c microglia in the molecular layer of dentate gyrus was observed 43 days after infection. At this point, only minor morphological changes were observed, and all the morphological changes induced by T. gondii infection at 22 dpi had disappeared. The analysis methods used included hierarchical cluster analysis (A), discriminant function analysis (C,E), morphological complexity (B), and convex hull volume (D).
